# Supplementary material for: Terahertz Néel spin-orbit torques drive nonlinear magnon dynamics in antiferromagnetic Mn2Au
Source: Nat Commun. 2023 Sep 27;14:6038. doi: 10.1038/s41467-023-41569-z (PMC10533548; doi:10.1038/s41467-023-41569-z)
Supplement: Supplementary file 1 — Supplementary Information [file 41467_2023_41569_MOESM1_ESM.pdf]

Supplementary Information

This Supplementary Information file contains

- Supplementary Note 1: Experimental Setup
- Supplementary Note 2: Terahertz-pulse characterization
- Supplementary Note 3: All signal combinations
- Supplementary Note 4: Spatial variation of $\Delta S(t)$ and nonvanishing $\langle \mathbf{L}_0 \rangle$
- Supplementary Note 5: Mn_2Au thin films with exchange-coupled permalloy cap layer
- Supplementary Note 6: Signal dependence on the driving field amplitude
- Supplementary Note 7: Possible nonmagnetic signal contributions
- Supplementary Note 8: Field-like Néel spin-orbit torques vs Zeeman torques
- Supplementary Note 9: Sample terahertz transmission and conductivity
- Supplementary Note 10: Estimation of transient temperature increase by terahertz pumping
- Supplementary Note 11: Mn_2Au films with thickness 25 nm
- Supplementary Note 12: Waveforms for various probe polarizations and sample rotations
- Supplementary Note 13: Detailed analysis of probe signal symmetry
- Supplementary references

Equations are numbered in extension of the main text, starting from Eq. (18), to facilitate cross-referencing.

Supplementary Note 1: Experimental Setup

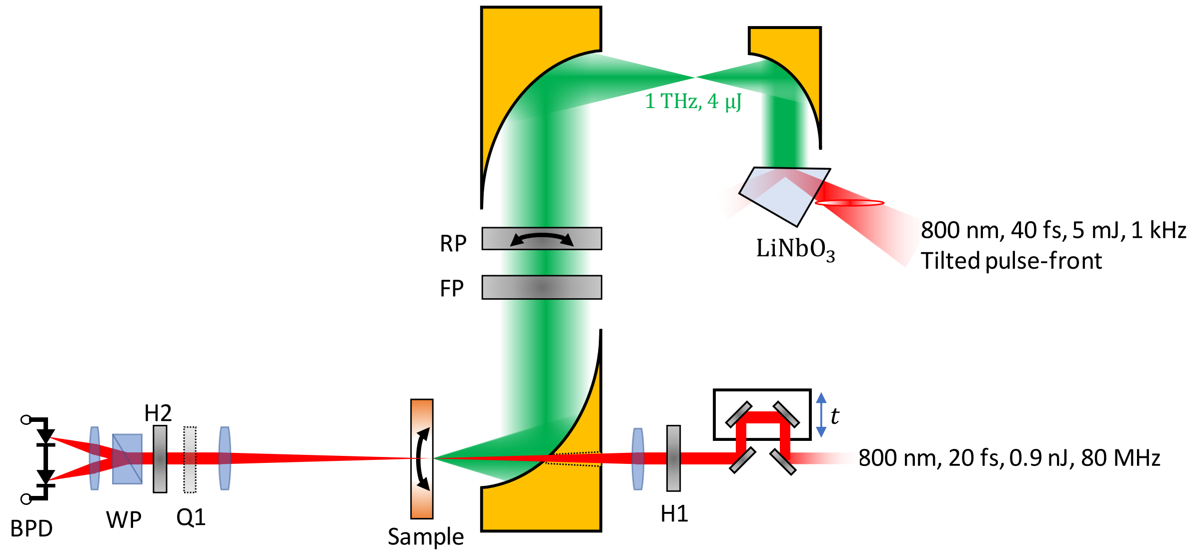

Fig. S1: Experimental setup. Terahertz pulses are generated by femtosecond laser pulses (center wavelength 800 nm, bandwidth 20 nm, duration 40 fs, repetition rate 1 kHz) from an amplified Ti:sapphire laser system through tilted pulse-front optical rectification in a $LiNbO_3$ crystal. The s-polarized terahertz pulses are guided to the sample by Au-coated off-axis parabolic mirrors. Each terahertz pulse passes a rotating wire-grid polarizer (RP) and a fixed polarizer (FP) as analyzer, the latter being transparent for p-polarized radiation. The terahertz field amplitude and polarity can be controlled by the orientation of the RP. The Mn_2Au sample is mounted on a rotation-translation stage that allows for rotation around the optical beam axis as well as translation of the sample. The linearly polarized probe pulses (center wavelength 820 nm, bandwidth 90 nm, duration 20 fs, repetition rate 80 MHz) are derived from the Ti:sapphire oscillator seeding the laser systems. They are delayed by a motorized stage with respect to the pump pulse, and their polarization state is controlled by a half-wave-plate (H1). Pump-induced changes in the polarization angle are detected by a combination of a half-wave plate (H2), Wollaston prism (WP) and balanced photodetector (BPD). To measure the ellipticity of the polarization, an additional quarter-wave plate (Q1) is inserted before H2.

Supplementary Note 2: Terahertz-pulse characterization

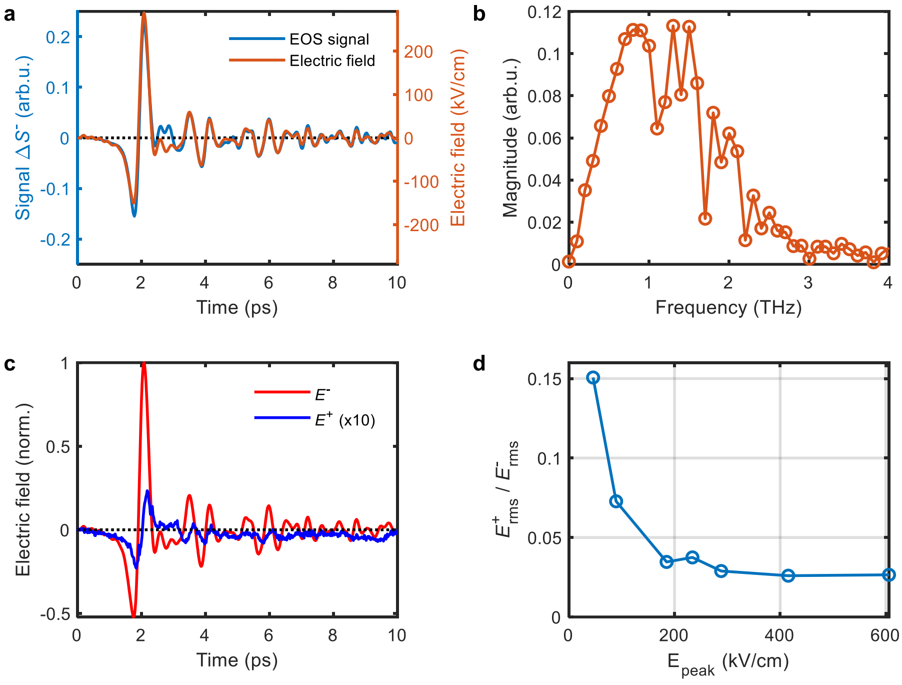

Fig. S2: Terahertz pulse characterization. **(a)** Blue line: Signal waveform ΔS^- (see text) obtained by electro-optic sampling in crystalline quartz (thickness 50 μm). It is odd with respect to the polarizer rotation angle α (RP in Fig. S1). Orange line: Extracted transient terahertz electric field $E^-(t)$ obtained by using the response function of the detection process. The peak field is calibrated by the independently measured terahertz power. The trailing features are attributed to rotational transitions in water molecules in the air. **(b)** Spectrum of the terahertz electric field of panel (a) as obtained by a Fourier transformation. Sharp features are due to extinction by residual water vapor. **(c)** Comparison of the components $E^-(t)$ and $E^+(t)$, respectively odd and even in the polarizer rotation angle α. Note that E^+ is multiplied by 10 for better visibility. **(d)** Ratio of the root-mean-square (rms) amplitudes of E^+ and E^- for various peak fields E_{pk} of the incident terahertz pulse.

We measure the transient electric field $\mathbf{E}(t)$ of the terahertz pulse in the focus by electro-optic sampling in a crystal of quartz (thickness of 50 μm) or ZnTe (10 μm). The resulting electro-optic signal $\Delta S(t)$ is related to the field component $E(t) = \mathbf{u}_{\mathrm{EO}} \cdot \mathbf{E}(t)$ along a certain axis $\mathbf{u}_{\mathrm{EO}}$ with $|\mathbf{u}_{\mathrm{EO}}| = 1$ by a transfer function. The component perpendicular to $\mathbf{u}_{\mathrm{EO}}$ does not contribute.

The terahertz field amplitude can be reversed using a rotating polarizer-analyzer geometry (see Supplementary Note 1), where the transmission axis of the rotating polarizer is set at angles $\pm\alpha$ with respect to the axis of the fixed polarizer FP. By varying the size of α, the transmitted terahertz field strength can also be controlled. Ideally, the resulting fields fulfill $\mathbf{E}(t, +\alpha) = -\mathbf{E}(t, -\alpha)$, leading to electro-optical signals $\Delta S(t, +\alpha) = -\Delta S(t, -\alpha)$. However, a small portion of the terahertz pulse passes the polarizer-analyzer configuration with a polarization along the incident polarization and, thus, cannot be controlled by the orientation of the polarizer. To quantify this leakage, we define even and odd contributions as $E^\pm = [E(t, +\alpha) \pm E(t, -\alpha)]/2$ and their electro-optic signals as $\Delta S^\pm = [\Delta S(t, +\alpha) \pm \Delta S(t, -\alpha)]/2$.

The measured electro-optic signal ΔS^- for quartz is shown in Fig. S2a (blue curve). While quartz provides good linearity up to amplitudes of ~ 1 MV cm^{-1}, its transfer function is frequency-dependent[1,2]. We, therefore, determine the temporal shape of the incident terahertz electric field from the measured signals by using a reference measurement with the ZnTe crystal, whose transfer function is known[3,4].

To determine the value of the peak field $E_{\mathrm{pk}} = \max|E(t)|$ independently, we measure the terahertz pulse energy W and intensity distribution of the terahertz intensity in the focus and make use of the relation

$$E_{\mathrm{pk}} = \sqrt{\frac{4\ln 2}{\pi}\frac{Z_0 W}{n\,\mathrm{FWHM}_I^2 \int \mathrm{d}t\, E^2(t)}}. \tag{18}$$

Here, $Z_0 \approx 377\ \Omega$ is the vacuum impedance, n is the refractive index of the material, and FWHM_I denotes the intensity full width at half maximum of the terahertz beam waist in the focus. The integration boundaries are chosen such that the major part of the intensity is captured. The waveform shown in Fig. S2 was taken for a terahertz pulse energy of $W = 500$ nJ, measured with a terahertz power sensor (Ophir 3A-P-THz) and a width $\mathrm{FWHM}_I = 950\ \mu$m, measured by placing a small aperture in the terahertz beam. This procedure yields a peak field strength of $E_{\mathrm{pk}} = 230$ kV cm^{-1} in air, consistent with estimates by the polarization change in electro-optic sampling. The extracted terahertz electric field is shown by the red curve in Fig. S2a, while its amplitude spectrum is displayed in Fig. S2b.

Fig. S2c displays the contributions E^- and E^+. As shown in Fig. S2d, the ratio of the amplitudes (root mean square) of E^+ and E^- is smaller than 5% at large fields and always better than 15%. This result demonstrates the high degree of terahertz polarization control with our setup. It implies that the terahertz electric-field component $\mathbf{u}_{\mathrm{EO}} \cdot \mathbf{E}$ along the sensitive direction $\mathbf{u}_{\mathrm{EO}}$ of the electro-optic detection can be considered as nearly fully reversing. However, this conclusion does not necessarily apply to the electric-field component perpendicular to $\mathbf{u}_{\mathrm{EO}}$, which is of the order of 5% for a single polarizer.

Supplementary Note 3: All signal combinations

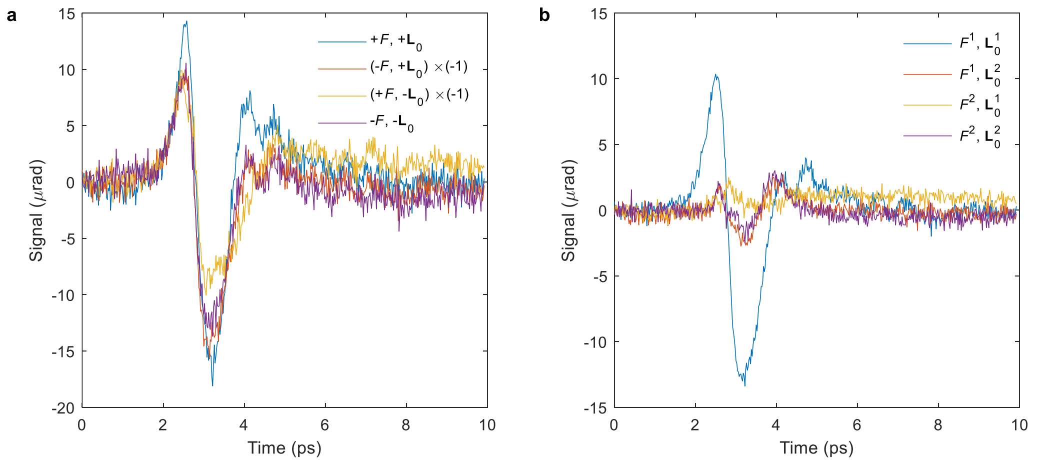

Fig. S3: **(a)** Birefringence signals for the combinations of electric field polarity F (controlled by wire-grid polarizers) and the local Néel vector $\pm\mathbf{L}_0$ (controlled by sample azimuth 0° vs 180°). For better comparison, red and yellow curves are reversed. **(b)** Signals separated in contributions odd and/or even in field polarity and sample rotation.

Fig. S3a shows the raw signals for 4 different experimental configurations, which are shorthand labeled $\pm F$ and $\pm\mathbf{L}_0$. For clarity, some signals are multiplied by -1. For $-F$, the signals reverse almost perfectly when $+\mathbf{L}_0 \rightarrow -\mathbf{L}_0$. However, for $+F$, a significant deviation from reversal is observed. A smaller deviation is also observed when we stay at $+\mathbf{L}_0$ and apply $+F \rightarrow -F$.

Fig. S3b shows the signals separated into odd and/or even parts with respect to F and $\mathbf{L}_0$. The separated contributions are determined according to

$$\begin{aligned} 4\Delta S(t, F^m, \mathbf{L}_0^n) = \quad & \Delta S(t, +F, +\mathbf{L}_0) + (-1)^m \Delta S(t, -F, +\mathbf{L}_0) \\ & + (-1)^n \, \Delta S(t, +F, -\mathbf{L}_0) + (-1)^{m+n} \Delta S(t, -F, -\mathbf{L}_0), \end{aligned} \tag{19}$$

where $m = 1, 2$ means odd (here linear) and even (here quadratic) in F, respectively, and $n = 1, 2$ is analogous, but with respect to $\mathbf{L}_0$. Eq. (19) is equivalent to the 2-step separation described in the main text.

The contribution $\Delta S(t, F^1, \mathbf{L}_0^1)$ odd in both F and $\mathbf{L}_0$, on which our study is focused, dominates over the other signals. However, for $\mathbf{L}_0^2$ (i.e., even in $\mathbf{L}_0$), the signals with F^2 (i.e., even in F) and F^1 (i.e., odd in F) have the same shape and significant amplitude. This observation might arise from (i) a process that is both odd and even in F at the same time or (ii) the imperfect reversal of F in our experimental approach based on field projections with 2 wire-grid polarizers (see Supplementary Note 2).

As scenario (i) is rather unlikely, we consider scenario (ii) and, thus, deal with terahertz electric fields of the form $\mathbf{E}_+(t) = E(t)\mathbf{u}_x + \epsilon(t)\mathbf{u}_y$ for $+F$ and $\mathbf{E}_-(t) = -E(t)\mathbf{u}_x$ for $-F$. In other words, $\mathbf{E}_+(t) + \mathbf{E}_-(t) = \epsilon(t)\mathbf{u}_y \neq 0$. Based on the transmission characteristics of the polarizers, the component ϵ is estimated to be of the order of 5% of the incident field. As a result, the Kerr effect in the sample substrate leads to an additional signal contribution that scales with $E\epsilon$. This component is allowed in all crystallographic symmetry groups and, because it is nonmagnetic, even in $\mathbf{L}_0$. Therefore, to fully extract the signal $\Delta S(t, F^1, \mathbf{L}_0^1)$, which contains the NSOTs contribution, both variations $\pm F$ and $\pm\mathbf{L}_0$ are required.

Supplementary Note 4: Spatial variation of $\Delta S(t)$ and nonvanishing $\langle \mathbf{L}_0 \rangle$

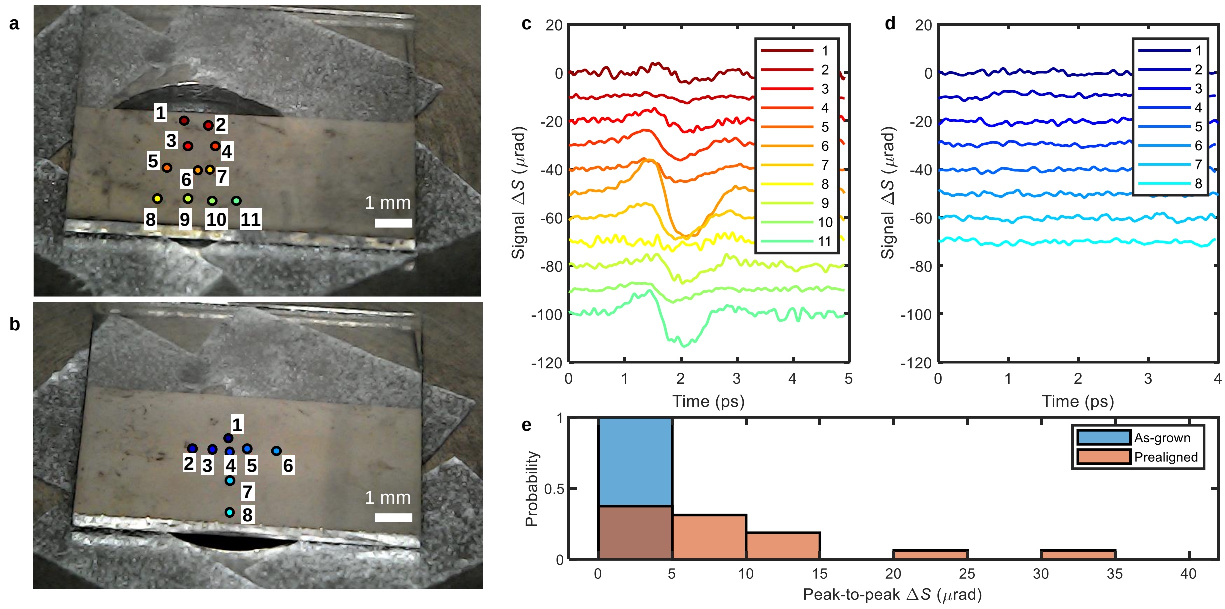

Fig. S4. Signal $\Delta S(t)$ odd in $\mathbf{E}$ and $\mathbf{L}_0$ for various spots on the prealigned [panels (a) and (c)] and as-grown sample [panels (b) and (d)]. **(a)** Probed spots 1-11 on the surface of the prealigned sample. The dimensions of the entire substrate are $10 \times 10 \times 0.5$ mm^3, and the sample film covers roughly half of its area. **(b)** Probed spots 1-8 on the surface of the as-grown sample. **(c)** Signals $\Delta S(t)$ for the probe spots 1-11 of the prealigned sample [panel (a)]. Signal traces are offset for clarity. **(d)** Same as panel (c), but for the spots 1-8 of the as-grown sample [panel (b)]. **(e)** Histogram of the peak-to-peak amplitudes of the signals on measured spots in the as-grown (blue) and prealigned sample (orange).

To explore the spatial structure of our samples, we investigate the signal on various and systematically chosen positions. We use a combination of rotation and translation stages to enable rotation around a fixed sample point, while also being able to choose the probed volume within a certain range of the sample surface. Figs. S4a and S4b show chosen points on a photograph for the prealigned and as-grown sample, respectively. Figs. S4c and S4d show the signals $\Delta S(t)$ that were measured on these points, with an individual offset for clarity.

Sizeable signals are only observed in the prealigned sample (Fig. S4c), whereas the signals of the as-grown sample are given by the experimental noise floor. In addition, Fig. S4c shows that the signal amplitude varies in a non-trivial manner over the sample surface, indicating a complex structure over distances much larger than the typical domain size in these samples. To emphasize the difference between prealigned and as-grown sample, Fig. S4e displays a histogram of the observed peak-to-peak signal amplitudes and demonstrates the much larger signal amplitudes observed in the prealigned sample.

As discussed in the methods, the signal $\Delta S(t)$ odd in the pump field and $\mathbf{L}_0$ scales with the Néel vector $\langle \mathbf{L}_0 \rangle$ averaged over the probe volume. We propose the following explanation of a nonvanishing $\langle \mathbf{L}_0 \rangle$ based on the prealignment procedure with the strong magnetic-field pulse $\mathbf{B}_{\text{ext}}$ (peak amplitude 60 T, duration 150 ms). While domain walls in an ideal antiferromagnet can move without changing the system energy, strain and defects tend to pin domain walls[5]. In the strong prealigning magnetic field, regions with $\mathbf{L}_0 \parallel \mathbf{B}_{\text{ext}}$, including domain walls, are energetically unfavorable and removed[6]. If the field is sufficiently large to overcome all domain-wall pinning energies, a single domain state is formed. However, if the field is smaller, multiple domains persist, but a favorable orientation of the Néel vector may still be expected in some regions. After the magnetic field is switched off, domain walls partially relax back, but some stay pinned due to local defects that cannot be overcome without aid of an external field, thus leaving a small remanence of the favored Néel vector direction.

The ratio $|\langle \mathbf{L}_0 \rangle| / |\mathbf{L}_0|$ is expected to be of the order of 1-10% (see Supplementary Note 5).

Supplementary Note 5: Mn_2Au thin films with exchange-coupled permalloy cap layer

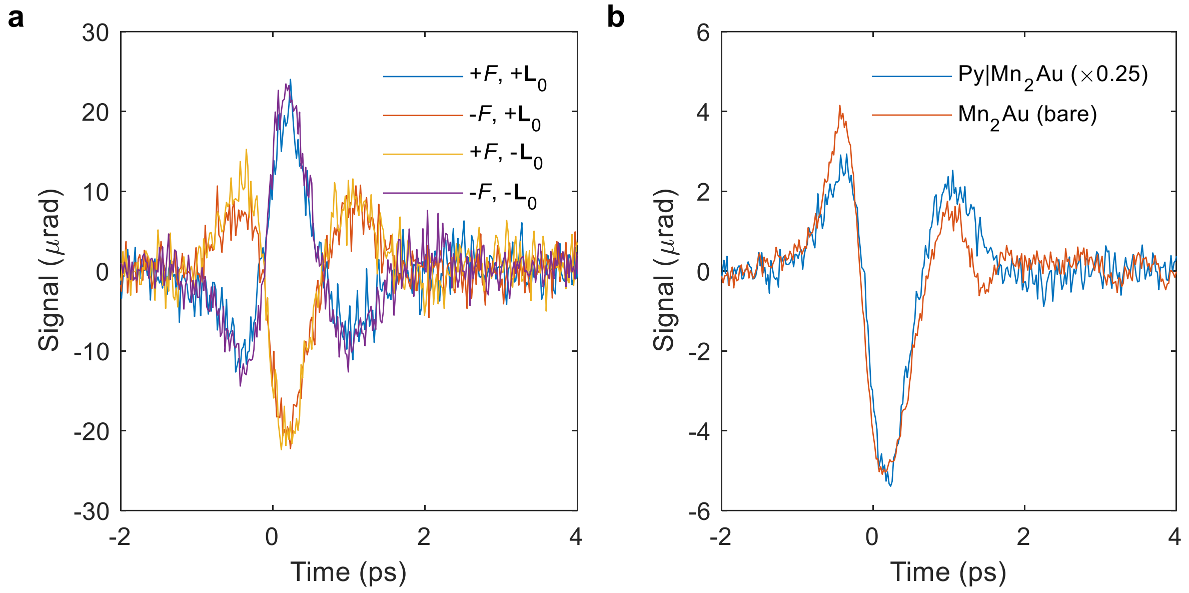

Fig. S5: Mn_2Au thin films with exchange-coupled permalloy (Py) cap layer **(a)** Magneto-optic signal waveforms from a stack MgO||Mo(23 nm)|Ta(10 nm)|Mn_2Au(50 nm)|Py(10 nm) grown on a MgO(500 µm) substrate for 4 combinations of positive/negative terahertz pump field ($\pm F$) and Néel-vector orientation ($\pm \mathbf{L}_0$). **(b)** Signals $\Delta S(t)$ odd in F and $\mathbf{L}_0$ from Mn_2Au|Py on MgO measured in reflection mode vs $\Delta S(t)$ from prealigned Mn_2Au on Al_2O_3.

To further confirm the magnetic origin of the pump-probe signal odd in F, we measure a control sample, MgO(500 µm)||Mo(23 nm)|Ta(10 nm)|Mn_2Au(50 nm)|Py(10 nm)|SiN(2 nm), where permalloy (Py) is shorthand for $Ni_{80}Fe_{20}$.Through exchange coupling, the equilibrium in-plane magnetization $\mathbf{M}_{\mathrm{Py}0}$ of the Py layer sets the probe-volume-averaged Néel vector $\langle \mathbf{L}_0 \rangle$ of the Mn_2Au layer[7], i.e., $\langle \mathbf{L}_0 \rangle \parallel \mathbf{M}_{\mathrm{Py}0}$. We use this effect to control the direction of $\mathbf{M}_{\mathrm{Py}0}$ and, thus, $\langle \mathbf{L}_0 \rangle$ by a moderate external magnetic field of 200 mT.

To reduce signal contributions from the Py top layer, the metal film is pumped and probed through the substrate side. To have sufficient probe power and access to beams, we measure in reflection mode with an angle of incidence of 55° and a p-polarized probe beam. The polarity of the terahertz field ($\pm F$) is controlled with a set of wire-grid polarizers. The experiment is performed under dry nitrogen atmosphere.

As seen in Fig. S5a, the signal waveforms are odd both in F and $\mathbf{L}_0$, consistent with our results from the magnetically prealigned Mn_2Au sample (Figs. 2a and 2b). The sign change with reversing $\mathbf{L}_0$ is a clear indication of the magnetic origin of the signal and corroborates our conclusion that a non-zero $\langle \mathbf{L}_0 \rangle$ is responsible for the observed signal.

Fig. S5b compares signals $\Delta S(t)$ odd in F and $\mathbf{L}_0$ from prealigned Mn_2Au on Al_2O_3 measured in transmission mode under normal incidence with $\Delta S(t)$ from Mn_2Au|Py on MgO measured in reflection mode. The signals agree well, and the signatures of the NSOT-induced mode are clearly visible. The small differences between the signal waveforms can be attributed to slightly altered terahertz pump pulses in the different measurement geometries.

Fig. S5b can also be used to estimate the domain imbalance in the bare Mn_2Au thin film. The peak terahertz electric fields of 15 kV cm^{-1} and 22 kV cm^{-1} inside the sample stacks are comparable for bare Mn_2Au and Mn_2Au|Py, respectively. The latter is probed in reflection geometry where the optical probe pulse needs to penetrate the substrate and seed layers, i.e., MgO(500 µm)||Mo(23 nm)|Ta(10 nm). Due to the seed layers and the reduced depth sensitivity in reflection, we estimate less efficient probing that reduces signals by a factor of 5. Assuming equal magneto-optic coefficients for reflected and transmitted beams and a single-domain state of the saturated Mn_2Au|Py samples, we can quantify the volume ratio $(0.5 + x)/(0.5 - x)$ of 0° vs 180° domains. We find that the signals from the bare

prealigned Mn_2Au film shown in Fig. S5b imply the order-of-magnitude estimate $x =$ 1-10%. This value agrees with the notion of a small statistic imbalance caused by the high magnetic-field pulses (see Supplementary Note 4).

Note that the control samples with Py are automatically reinitialized by the external magnetic field in between consecutive terahertz pump pulses. As they show almost identical behavior compared to the bare Mn_2Au films (Fig. S5b), we conclude that the bare samples return to their initial state following excitation. This conclusion is reasonable because in the hypothetical case of permanent switching, the NSOTs would be zero ($\mathbf{E} \cdot \mathbf{L}_0 = 0$), and the pump-probe signal would disappear. To summarize, the observed dynamics are fully reversible without any permanent switching.

Supplementary Note 6: Signal dependence on the driving field amplitude

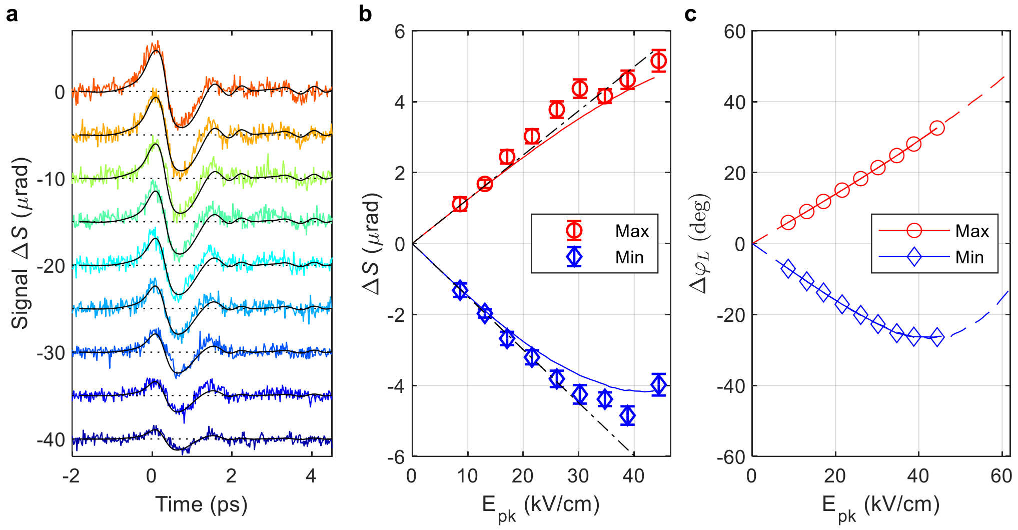

Fig. S6: Field dependence of the signal traces $\Delta S(t)$ odd in the terahertz field F and the equilibrium Néel vector $\mathbf{L}_0$ for the data points given in Fig. 2d. **(a)** Measured waveforms for various terahertz peak electric fields E_{pk} inside Mn_2Au. Black lines are model calculations based on Eq. (17) and the parameters obtained from Fig. 4b, where only the signal amplitude was rescaled. The rescaling arises from averaging of 0° vs 180° domains in the probed region (see Methods) compared to the one shown in Fig. 4. Signals are vertically offset for clarity, and the gray-dotted lines indicate the respective zero value. **(b)** Maximum (blue circles) and minimum (red diamonds) signal amplitude vs peak electric field E_{pk} inside the sample (same as Fig. 2c). The blue/red solid line shows the calculated maximum/minimum amplitude given by the black lines in panel (a). The error bars are calculated from the standard deviation of the first 40 data points per trace in panel (a). The dash-dotted black lines are the linear approximations of the model. A nonlinear response emerges at fields $E_{\mathrm{pk}} > 30$ kV cm^{-1} inside the sample, primarily seen in the saturation of the minimum amplitude. **(c)** Corresponding Néel vector deflection for the signals shown in panel (a) and extrapolation to larger fields. For fields above 30 kV cm^{-1}, the maximum deflection keeps rising, while the minimum amplitude levels off and subsequently decreases due to the nonlinear spin dynamics [Eq. (17)] and nonlinear magneto-optic response [Eq. (16)].

Supplementary Note 7: Possible nonmagnetic signal contributions

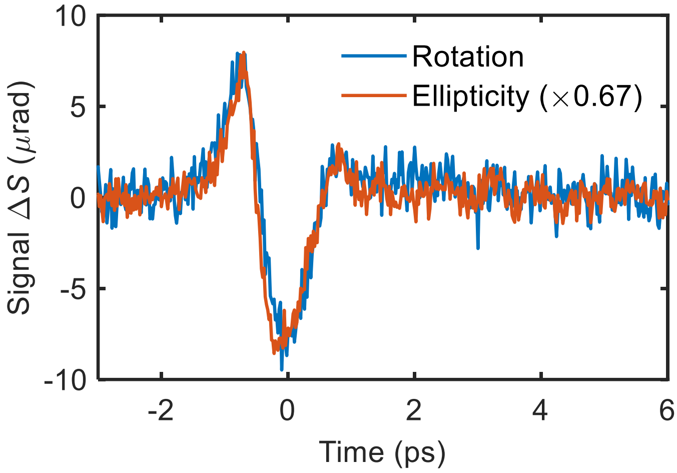

Fig. S7: Signals of probe polarization rotation (blue line) and ellipticity (red) signals odd in both F and $\mathbf{L}_0$ obtained from the prealigned Mn_2Au sample on a $Al_2O_3(1\bar{1}02)$ substrate plus an identical yet 90°-rotated $Al_2O_3(1\bar{1}02)$ substrate behind it.

According to Eq. (2), the measured pump-probe signal contains, in principle, a contribution $\Delta S_{\mathcal{N}}$ that is not related to any transient changes in $\mathbf{L}$ and $\mathbf{M}$. It exclusively arises from variations of the non-spin degrees of freedom $\mathcal{N}$ of Mn_2Au and can be shown to exhibit the same dependence on $\varphi_{\mathbf{E}}$, $\varphi_{\mathbf{L}_0}$ and φ_s as the a, b and c terms of Eq. (2) (see Methods and Supplementary Note 13). However, we do not expect $\Delta S_{\mathcal{N}}$ to make a dominant contribution to the total signal because of the following reasons.

First, we measure pump-induced changes $\Delta S(t)$ odd in both terahertz field F and Neel vector $\mathbf{L}_0$ for both the polarization rotation and ellipticity of the probe beam. The 2 signals exhibit the same dynamics (Fig. S7), as dictated by the first 3 terms of Eq. (2). In contrast, $\Delta S_{\mathcal{N}}$ does not require identical evolution of probe rotation and ellipticity.

Second, pump-induced electron orbital dynamics linear in the driving electric field are almost instantaneous in metals. In particular, in Mn_2Au, the terahertz-field-driven electron current decays in < 50 fs according to terahertz conductivity measurements (see Supplementary Note 9). It is, thus, unlikely to be linked to the much slower measured response (Fig. 2d).

Third, we exclude significant crystal-lattice dynamics because terahertz transmission experiments do not indicate long-wavelength phonons over our pump-pulse spectrum[8]. Other metallic antiferromagnets exhibit their lowest optical-phonon frequencies between 3 THz and 6 THz,[9-11] significantly faster than the response observed here.

Finally, as discussed in the main text, our measured signals can be well explained by pure spin dynamics.

Supplementary Note 8: Field-like Néel spin-orbit torques vs Zeeman torques

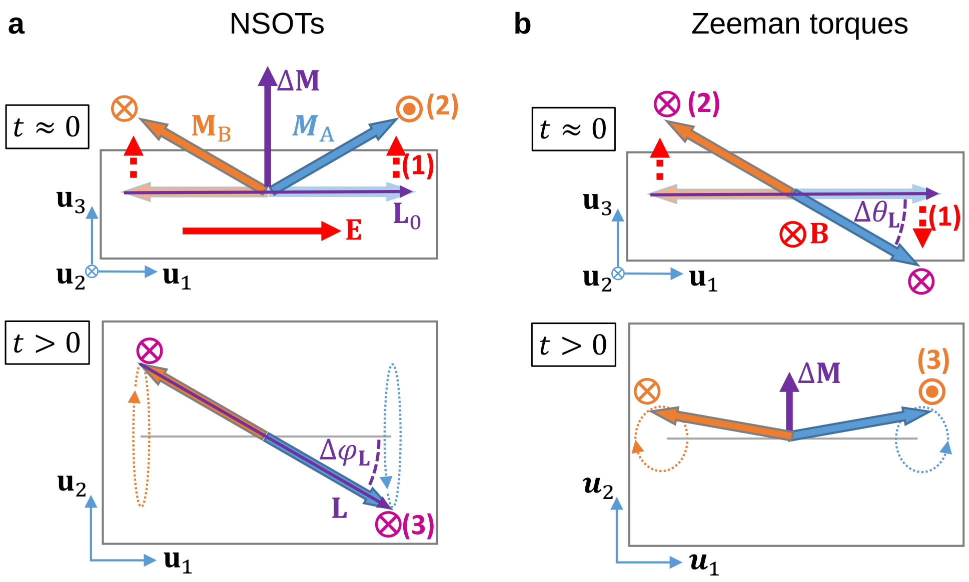

Fig. S8: Field-like Néel spin-orbit torques vs Zeeman torques in Mn_2Au for impulsive excitation. **(a)** Dynamics driven by NSOTs (also described in Fig. 3). (1) The impulsive electric field $\mathbf{E}(t) \propto \delta(t)$ leads to staggered spin-orbit fields and equal torques on the sublattices (red-dashed arrows). The resulting out-of-plane magnetization $\Delta\mathbf{M} \parallel \mathbf{u}_3$, in turn, leads to exchange torques with opposite sign (orange symbols). (2) A strong in-plane deflection of the Néel vector $\perp \mathbf{u}_3$ ensues and, along with the *in-plane* anisotropy field $B_{\mathrm{ani}} \ll B_{\mathrm{ex}}$, induces equal torques[12]. The resulting precession of the sublattices is strongly elliptical with the major axis being $\parallel \mathbf{u}_2$. Consequently, the only non-zero components of the order parameters in the linear regime are $L_1(t) = \mathbf{u}_1 \cdot \mathbf{L}(t) \approx \mathbf{u}_1 \cdot \mathbf{L}_0 =: L_0$, $L_2(t) \approx L_0 \Delta\varphi_{\mathrm{L}}(t)$ and $M_3(t) = \mathbf{u}_3 \cdot \mathbf{M}(t)$. **(b)** Dynamics driven by Zeeman torque through an impulsive magnetic field $\mathbf{B}(t) \propto \delta(t)$. (1) The resulting torques are opposite for the 2 sublattices, in contrast to NSOTs [panel (a)]. They induce an out-of-plane deflection $L_3 \approx L_0 \Delta\theta_{\mathrm{L}}$ of the Néel vector. (2) As the sublattices are still parallel, no exchange torques are active. The only torques derive from the uniaxial (out-of-plane) anisotropy because $\mathbf{u}_3$ points along a magnetically hard axis. The resulting torques are $\propto |\mathbf{B}_{\mathrm{ani}\parallel}|$ and of equal sign, leading to a canting along $\mathbf{u}_2$, which (3) finally triggers opposite exchange torques (orange symbols). The resulting precession of the sublattices is again elliptical, but the major axis is $\parallel \mathbf{u}_3$. The non-zero components of the order parameters in the linear regime are $L_1(t) \approx L_0$, $L_3(t) \approx L_0 \Delta\theta_{\mathrm{L}}(t)$ and $M_2(t) = \mathbf{u}_2 \cdot \mathbf{M}(t)$.

As shown in Supplementary Note 13, the symmetry of our experiment does not allow for the detection of effects linear in the terahertz magnetic field, e.g., due to Zeeman torque. However, magnetic fields (and their time-derivatives) can certainly excite magnon modes in antiferromagnets[13-15]. Here, we briefly discuss the excitation of such a mode in Mn_2Au.

Fig. S8a (like Fig. 3a) shows the excitation process for an impulsive electric field in the sample plane spanned by $\mathbf{u}_1$ and $\mathbf{u}_2$. Because of exchange enhancement, the maximum $M_3 = \mathbf{u}_3 \cdot \mathbf{M}$ is orders of magnitude smaller than the maximum $L_2 = \mathbf{u}_2 \cdot \mathbf{L}$. Therefore, the mode can be called in-plane ($\perp \mathbf{u}_3$), and its angular frequency is given by $\Omega_0 = 2\gamma\sqrt{B_{\mathrm{ex}} B_{\mathrm{ani}}}$.

For the excitation with an impulsive magnetic field (Fig. S8b), the situation is quite different. We focus on an in-plane field, which is most relevant for our terahertz pump pulse. As Zeeman torque ($\propto \mathbf{B}$) and field-derivative torque ($\propto \partial\mathbf{B}/\partial t$) have the same symmetry, the discussion applies to both cases. As detailed in Fig. S8b, $\mathbf{B}$ induces opposite torques and, consequently, excites a different mode than NSOTs. Exchange enhancement is still present, but the mode is governed by the *out-of-plane* anisotropy field $B_{\mathrm{ani}\parallel}$ and has angular frequency[12] $\Omega_1 \approx \gamma\sqrt{2B_{\mathrm{ex}} B_{\mathrm{ani}\parallel}}$.

From magnetic-susceptibility measurements[16], it was concluded that $B_{\mathrm{ani}\parallel} \gg B_{\mathrm{ani}}$, and Raman-scattering studies[17] indicated a mode with $\Omega_1/2\pi \approx 3.7$ THz $> \Omega_0/2\pi$. The exchange-enhanced deflection leads to a sizeable $L_3(t)$ and small $M_2(t)$. Comparison with Eq. (9) and (39), however, shows that (i) the corresponding signals would be even in $\mathbf{L}_0$ and (ii) the only detectable signal

component in our experimental geometry would be $\propto (\mathbf{u}_y \cdot \Delta\mathbf{M})^2$. The latter is very small compared to the signal proportional to the deflection $\Delta\mathbf{L}$ of the Néel vector.

We note that the low-frequency mode (Fig. S8a) can in principle also be excited by a time-dependent magnetic field $\parallel \mathbf{u}_3$. However, such fields are zero in the center of Gaussian beams, making an experimental verification challenging.

Supplementary Note 9: Terahertz transmission and conductivity

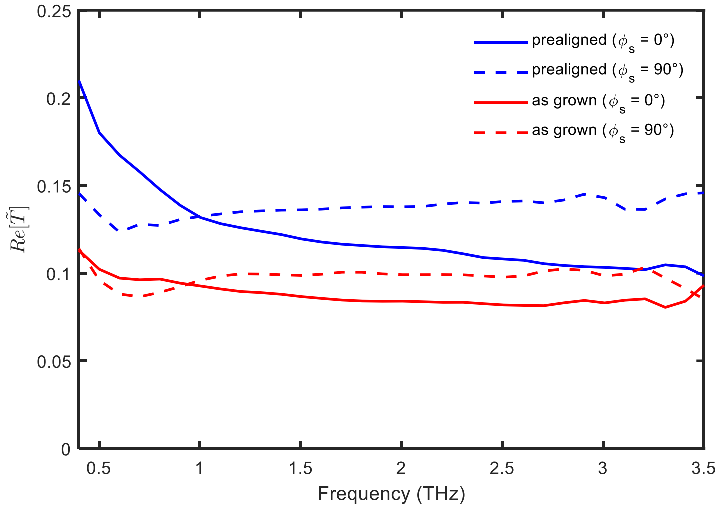

Fig. S9: Terahertz thin-film amplitude transmission coefficient vs frequency. Blue curves refer to the prealigned sample for $\varphi_s = 0°$ and 90° (Fig. 1a) as solid and dashed lines, respectively. Red curves refer to the as-grown sample for $\varphi_s = 0°$ and 90° as solid and dashed lines, respectively.

To estimate the terahertz conductivity and, thus, the terahertz field strength inside the sample, we measure the transmission of the Mn_2Au thin film in a separate terahertz time-domain spectroscopy experiment[18]. More precisely, we compare the terahertz pulse transmitted through (i) the bare Al_2O_3-substrate (sub) and (ii) the substrate with the Mn_2Au thin film (Mn_2Au|sub, Fig. 1a). The amplitude transmission coefficient at frequency $\omega/2\pi$ is in the thin-film approximation given by[19]

$$\tilde{T}(\omega) = \frac{\tilde{E}_{\mathrm{Mn_2Au|sub}}(\omega)}{\tilde{E}_{\mathrm{sub}}(\omega)} = \frac{n_{\mathrm{a}}(\omega) + n_{\mathrm{sub}}(\omega)}{n_{\mathrm{a}}(\omega) + n_{\mathrm{sub}}(\omega) + Z_0 G(\omega)}, \tag{20}$$

where n_{a} and n_{sub} is the refractive index of air and substrate, respectively, Z_0 is the vacuum impedance, and G is the conductance of the Mn_2Au thin film.

Fig. S9 shows $\tilde{T}(\omega)$ for prealigned and as-grown sample for 2 sample orientations $\varphi_s = 0°$ and 90° (Fig. 1a). We find that the transmission coefficient $\tilde{T}(\omega)$ behaves similarly for prealigned and as-grown sample, with a somewhat smaller transmission for the as-grown sample.

Note that the denominator in Eq. (20) is dominated by the metal-film conductance through $Z_0 G(\omega)$, whereas the nominator is more sensitive to the birefringence of the substrate. At 1 THz, the $Al_2O_3(1\bar{1}02)$ substrate exhibits a static birefringence with a refractive index[20] of, respectively, 3.1 and 3.4 for the ordinary and extraordinary ray. The resulting offset is consistent with the measured $\tilde{T}(\omega)$ for each sample measured at $\varphi_s = 0°$ vs 90° (Fig. S9).

From Eq. (20), the film conductance can be calculated as

$$G(\omega) = \frac{n_{\mathrm{a}}(\omega) + n_{\mathrm{sub}}(\omega)}{Z_0}\left[\frac{1}{\tilde{T}(\omega)} - 1\right], \tag{21}$$

where we use[21] $n_{\mathrm{a}} = 1$, $n_{\mathrm{sub}} = n_{\mathrm{Al_2O_3,o}} = 3.08$ and $Z_0 = 377\ \Omega$. We find a conductance of 75 mS and 100 mS for the, respectively, prealigned and as-grown samples, when averaging over multiple positions on the sample. With the film thickness of $d = 50$ nm, a mean conductivity $\sigma = G/d$ of 1.5 MS m^{-1} and 2 MS m^{-1} is obtained for the prealigned and as-grown sample, respectively. These values are consistent with the values of 2 MS m^{-1} and 3.6 MS m^{-1} obtained with a DC 4-point-probe measurement. We attribute the overall lower terahertz conductivity to its higher sensitivity with respect to the local sample structure and the birefringent substrate.

Using these measurements and Eq. (20), we can calculate the electric field strength $\tilde{E}(\omega)$ *inside* the Mn_2Au sample film relative to the incident field $\tilde{E}_{\mathrm{inc}}(\omega)$ as

$$\frac{\tilde{E}(\omega)}{\tilde{E}_{\text{inc}}(\omega)} = \frac{2n_{\text{a}}(\omega)}{n_{\text{a}}(\omega) + n_{\text{sub}}(\omega)}\tilde{T}(\omega) = \frac{2n_{\text{a}}(\omega)}{n_{\text{a}}(\omega) + n_{\text{sub}}(\omega) + Z_0 G(\omega)}. \quad (22)$$

For an average transmission of $\tilde{T}(\omega)$ of 0.13, we obtain that the field inside the sample relative to the incident field is $\tilde{E}(\omega) = 6\%\ \tilde{E}_{\text{inc}}(\omega)$. The dominance of $Z_0 G \approx 30$ in the denominator of Eq. (22) implies that the refractive-index anisotropy of the substrate has a minor impact on the terahertz electric field inside the metal film.

In principle, the terahertz transmission spectra should also contain a signature of the terahertz-NSOTs-driven spin dynamics. A conservative estimate based on the inverse-NSOTs mechanism[12] suggests a variation of the terahertz field transmission of the order of 1% between the configurations $\mathbf{E} \parallel \mathbf{L}_0$ and $\mathbf{E} \perp \mathbf{L}_0$. However, this effect is too small to be clearly visible in our terahertz transmission spectra (Fig. S9). Its isolation requires dedicated modulation schemes[19].

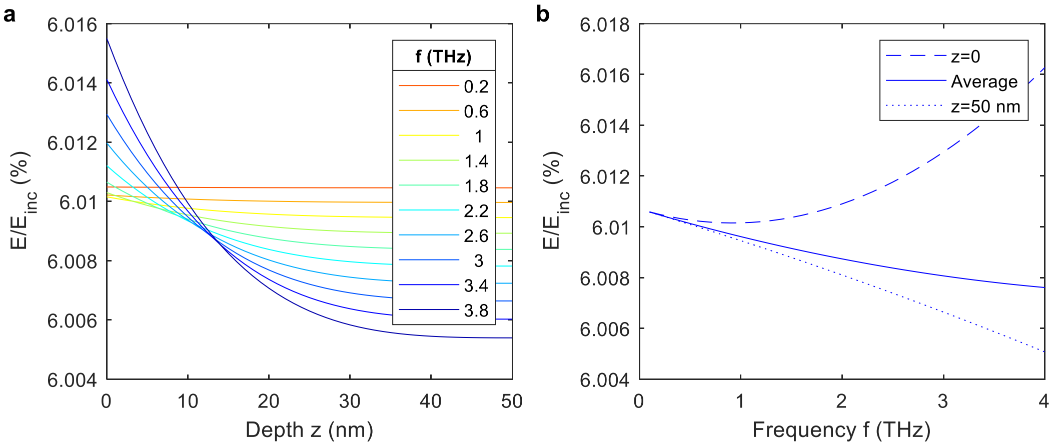

Fig. S10: Terahertz electric-field distribution inside the 50 nm Mn_2Au thin film. **(a)** $|\tilde{E}(z,\omega)/\tilde{E}_{\text{inc}}(\omega)|$ vs depth z for a terahertz pulse impinging from free space ($z < 0$). Colored solid lines correspond to components with different frequency $f = \omega/2\pi$. **(b)** Terahertz electric field at the air/sample ($z = 0$) and sample/substrate boundary ($z = 50$ nm), as well as averaged over the full thickness of the sample film vs frequency. Note the ordinate scaling.

To confirm the validity of the thin-film approximation underlying Eq. (20), we numerically calculate the terahertz electric-field distribution inside the 50 nm thick Mn_2Au film using a transfer-matrix formalism[22] and assuming a frequency-independent conductivity. Fig. S10a shows the normalized terahertz electric field $|\tilde{E}(z,\omega)/\tilde{E}_{\text{inc}}(\omega)|$ vs depth coordinate z for various frequencies $\omega/2\pi$, where $E_{\text{inc}}(\omega)$ is the incident field amplitude at $z = 0$. The field amplitude is constant over the full film thickness to very good approximation and amounts to 6% of E_{inc}. This result is in good agreement with the thin-film formula and holds well beyond the bandwidth of the terahertz pulse in our experiment.

Fig. S10b shows the field amplitude at $z = 0$ and $z = 50$ nm as well as the z-averaged field inside the sample, which differ negligibly up to 4 THz and beyond. This result is consistent with the skin depth $\sqrt{2c/Z_0\sigma(\omega)\omega} \approx 400$ nm at 1 THz, where c and Z_0 are, respectively, the speed of light and impedance of free space. Effects of field gradients in the sample excitation can, therefore, be neglected.

Supplementary Note 10: Estimation of transient temperature increase by terahertz pumping

The absorbed energy per unit volume in the Mn_2Au thin film is given by $\partial W_{\mathrm{abs}}/\partial V = \int \mathrm{d}t\, \mathbf{j}(t) \cdot \mathbf{E}(t)$, where $\mathbf{E}(t)$ is the electric field inside the film. Using $\mathbf{j}(t) = \sigma \mathbf{E}(t)$ and the average terahertz conductivity $\sigma = 1.5$ MS m^{-1} (Supplementary Note 9), we obtain $\partial W_{\mathrm{abs}}/\partial V = \sigma \int \mathrm{d}t\, \mathbf{E}^2(t) \approx 7$ J cm^{-3}.

The $\partial W_{\mathrm{abs}}/\partial V$ can be related to a temperature increase $\Delta T = (\partial W_{\mathrm{abs}}/\partial V)/c_p$, where c_p is the specific heat capacity of Mn_2Au, which can be estimated as $c_p \approx \left(2c_p^{\mathrm{Mn}} + c_p^{\mathrm{Au}}\right)/3 \approx 0.363$ J g^{-1} K^{-1}, with the constituent values taken from Ref. [23] at room temperature. Estimation of the mass density of Mn_2Au by the atomic mass and unit-cell size yields $\rho = 10.93$ g cm^{-3}, and we obtain a temperature increase of $\Delta T \approx 1.7$ K for the largest available terahertz fluence of a single terahertz pulse.

With the predicted field strength $\sim 2.5E_0$ required for switching (Fig. 4d), the fluence increases 6-fold, leading to an estimated temperature increase of 10 K. Therefore, NSOTs should allow for cold switching at terahertz frequencies.

Supplementary Note 11: Mn_2Au films with thickness 25 nm

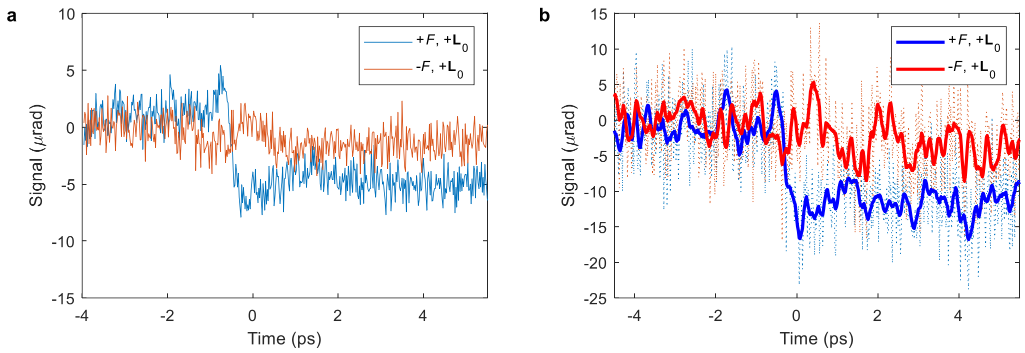

Fig. S11: Transient birefringence signals for as-grown and prealigned 25 nm thick Mn_2Au films. **(a)** As-grown film. Blue and orange lines are the signals for configurations $\pm F$, respectively. **(b)** Prealigned film. The blue/red dotted lines are raw data for configuration $\pm F$, respectively, whereas solid lines are smoothed. The lower signal-to-noise ratio compared to panel (a) stems from a 10 times shorter averaging time in these measurements.

As a further check, we studied films with a significantly smaller thickness of 25 nm (Fig. S11). We find that both as-grown and prealigned sample show similar signals. Notably, only one configuration ($+F$) contains a significant step-like response. As discussed in Supplementary Note 3, this step likely originates from the Kerr effect in the substrate plus an imperfect reversal of the terahertz field $\pm F$, since the fields of the "$+F$" and "$-F$" configurations are not exactly reversed version and antiparallel versions of each other.

The resulting signal effect scales with F^2 and, thus, $1/d^2$, where d is the metal-film thickness (see Supplementary Note 9). Consequently, the Kerr effect is much more pronounced in thinner films and makes the study of spin-dynamics signals challenging. If the magnetic signal is still present in the 25 nm sample on top of the strong Kerr response, it must be about 1 order of magnitude smaller than in the 50 nm samples. This notion is consistent with a larger relative role of an interfacial MnAu impurity phase in thinner Mn_2Au films (see Methods and Ref.[24]), where the impurity phase is not expected to feature the NSOTs present in epitaxial Mn_2Au.

Supplementary Note 12: Waveforms for various probe polarizations and sample rotations

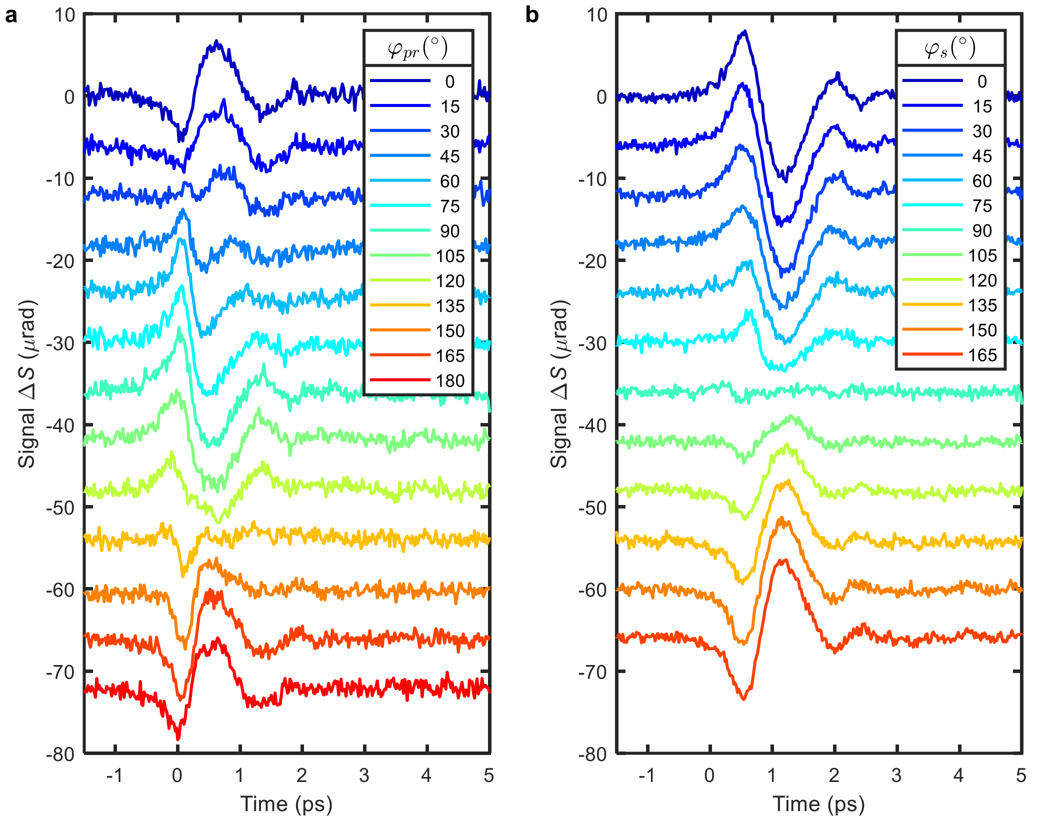

Fig. S12: Terahertz signal waveforms vs probe polarization and azimuthal sample rotation **(a)** Signal waveforms $\Delta S(F^1, +\mathbf{L}_0)$ odd in the pump field $F = (\mathbf{E}, \mathbf{B})$ for different incident probe polarization angles φ_{pr}, corresponding to Figs. 2e and 2f of the main text. The sample azimuth is kept at $\varphi_s = 0°$. **(b)** Signal waveforms $\Delta S(F^1, \mathbf{L}_0^1)$ odd in both F and $\mathbf{L}_0$ for different sample angles φ_s, corresponding to Figs. 3b and 3c of the main text. In this configuration, simultaneous rotation of the sample and probe polarization guarantees $\varphi_{\mathrm{pr}} = \varphi_s$.

Supplementary Note 13: Detailed analysis of probe signal symmetry

In this note, we give a detailed analysis of the probe-signal symmetry based on Eq. (6) of the Methods section and the subsequent discussion.

Optical conductivity tensor

All quantities in Ohm's law [Eq. (5) of the Methods] refer to a given Cartesian coordinate system Σ. Therefore, $\mathbf{j}_{\mathrm{pr}}$ and $\mathbf{E}_{\mathrm{pr}}$ are columns, and $\underline{\sigma}$ is a square matrix. We now apply a (proper or improper) rotation to Σ to obtain another Cartesian coordinate system Σ'. In short, we have the transformation

$$\Sigma \xrightarrow{\underline{R}} \Sigma', \tag{23}$$

where the unitary matrix $\underline{R}$ describes the rotation. Improper rotations ($\det \underline{R} = -1$) can be understood as proper rotations ($\det \underline{R} = +1$) combined with reflections of 1 or 3 selected coordinate axes. In the primed frame Σ', Ohm's law [Eq. (5)] becomes

$$\mathbf{j}'_{\mathrm{pr}} = \underline{\sigma}'\mathbf{E}'_{\mathrm{pr}}. \tag{24}$$

Because $\mathbf{j}_{\mathrm{pr}}$ and $\mathbf{E}_{\mathrm{pr}}$ are polar vectors, they transform according to $\mathbf{j}'_{\mathrm{pr}} = \underline{R}^{-1}\mathbf{j}_{\mathrm{pr}}$ and $\mathbf{E}'_{\mathrm{pr}} = \underline{R}^{-1}\mathbf{E}_{\mathrm{pr}}$. Combination with Eqs. (5) and (24) results in

$$\underline{\sigma}' = \underline{R}^{-1}\underline{\sigma}\underline{R}. \tag{25}$$

Eq. (25) quantifies how the conductivity tensor (matrix) transforms when we change the coordinate system according to Eq. (23). In the specific case of space inversion $\underline{R} = -\mathbb{I} = \underline{R}^{-1}$, Eq. (25) implies $\underline{\sigma}' = \underline{\sigma}$. In other words, the electrical conductivity tensor is invariant under space inversion regardless of the sample symmetry.

The "passive" view of rotating the coordinate system according to Eq. (23) can be replaced by an equivalent "active" interpretation, which is often helpful for symmetry considerations. In this view, Eq. (25) describes how the conductivity tensor changes when the sample and all the objects that belong to it are rotated in the inverse manner according to

$$\text{sample} \xrightarrow{\underline{R}^{-1}} \text{sample}'. \tag{26}$$

Sample state

We note that the state and, thus, conductivity of the sample are fully determined by a sufficiently large tuple $\mathcal{X} = (X_1, X_2, \ldots)$ of observables in Σ and, likewise, $\mathcal{X}' = (X'_1, X'_2, \ldots)$ in Σ'. These observables include all sample degrees of freedom together with external electromagnetic fields such as that of the terahertz pump pulse. Therefore, we can symbolically write

$$\underline{\sigma} = f(\mathcal{X}) \text{ and } \underline{\sigma}' = f(\mathcal{X}'), \tag{27}$$

where f is a suitable function (or functional) mapping $\mathcal{X}$ on $\underline{\sigma}$. Importantly, when the operation $\underline{R}$ is a symmetry operation that leaves the sample invariant ($\text{sample}' = \text{sample}$), we have $\mathcal{X}' = \mathcal{X}$ and, thus, $\underline{\sigma}' = \underline{\sigma}$.

For Mn_2Au, a possible choice for $\mathcal{X}$ is

$$\mathcal{X} = (\mathcal{N}, \mathcal{S}). \tag{28}$$

It separates $\mathcal{X}$ in spin-related degrees of freedom $\mathcal{S}$ and the other, non-spin degrees of freedom $\mathcal{N}$. Examples of observables contained in $\mathcal{N}$ are the positions of all electrons and all nuclei of the crystal lattice. For our purposes, we assume that the spin system is sufficiently characterized by the local sub-lattice magnetizations $\mathbf{M}_1$ and $\mathbf{M}_2$, implying $\mathcal{S} = (\mathbf{M}_1, \mathbf{M}_2)$. Equivalently, one can also use $\mathcal{S} = (\mathbf{M}, \mathbf{L})$ with total magnetization $\mathbf{M} = \mathbf{M}_1 + \mathbf{M}_2$ and Néel vector $\mathbf{L} = \mathbf{M}_1 - \mathbf{M}_2$.

The impact of the pump pulse on the sample is fully captured by the time-dependent state characteristics $\mathcal{N}(t) = \mathcal{N}_0 + \Delta\mathcal{N}(t)$ and $\mathcal{S}(t) = \mathcal{S}_0 + \Delta\mathcal{S}(t)$, where $\mathcal{N}_0$ and $\mathcal{S}_0$ refer to the stationary sample before arrival of the pump. The time-dependent changes $\Delta\mathcal{N}(t)$ and $\Delta\mathcal{S}(t)$ are determined by the pump field and the initial sample state $(N_0, \mathcal{S}_0)$. Therefore, one can alternatively choose

$$\mathcal{X} = (\mathcal{N}_0, \mathcal{S}_0, F), \tag{29}$$

where $F = (\mathbf{E}, \mathbf{B})$ summarizes the electric and magnetic component $\mathbf{E}$ and $\mathbf{B}$ of the pump field.

Eqs. (28) and (29) consider the action the pump field implicitly and explicitly, respectively. While the choice of Eq. (29) yields information on how the pump field modifies the probe response, the choice of Eq. (28) helps us understand how the instantaneous magnetic order $\mathcal{S}(t)$ modifies the probe signal. In the following, we will discuss these 2 viewpoints in more detail.

Pump field: explicit treatment

Space inversion. We start with considering the impact of the pump field on the probe response [see Eq. (29)] and first focus on space inversion $\underline{R} = -\mathbb{I}$. In this case, Eqs. (25) and (27) imply $f(\mathcal{X}') = f(\mathcal{X})$, which can be written in the shorter yet slightly ambiguous form

$$\underline{\sigma}(\mathcal{X}') = \underline{\sigma}(\mathcal{X}). \tag{30}$$

To analyze the transformation of $\mathcal{X} = (\mathcal{N}_0, \mathcal{S}_0, F)$ [see Eq. (29)] under space inversion, we note that the pump field $F = (\mathbf{E}, \mathbf{B})$ turns into $F' = (-\mathbf{E}, \mathbf{B})$ because $\mathbf{E}$ is a polar vector, whereas $\mathbf{B}$ is axial. Owing to the symmetry properties of Mn_2Au, space inversion leaves the unperturbed non-spin degrees of freedom invariant: $\mathcal{N}_0' = \mathcal{N}_0$. It, however, swaps the roles of the 2 spin-sublattices and, thus, turns $\mathcal{S}_0 = (\mathbf{M}_{10}, \mathbf{M}_{20})$ into $\mathcal{S}_0' = (\mathbf{M}_{10}', \mathbf{M}_{20}') = (\mathbf{M}_{20}, \mathbf{M}_{10})$. Equivalently, one can say that the total equilibrium magnetization $\mathbf{M}_0 = \mathbf{M}_{10} + \mathbf{M}_{20}$ remains invariant, whereas the sign of the Néel vector $\mathbf{L}_0 = \mathbf{M}_{10} - \mathbf{M}_{20}$ reverses. As a consequence, the (improper) rotations can be further divided into staggered operations (changing the sublattice role) and non-staggered operations[25].

To summarize, the impact of space inversion and Eq. (30) result in

$$\underline{\sigma}(\mathcal{N}_0, \mathbf{L}_0, \mathbf{E}, \mathbf{B}) = \underline{\sigma}(\mathcal{N}_0, -\mathbf{L}_0, -\mathbf{E}, \mathbf{B}), \tag{31}$$

where we omitted $\mathbf{M}_0 = 0$ in the notation. Eq. (31) implies that the response odd in $\mathbf{E}$ [i.e., $\underline{\sigma}(\mathcal{N}_0, \mathbf{L}_0, \mathbf{E}, \mathbf{B}) - \underline{\sigma}(\mathcal{N}_0, \mathbf{L}_0, -\mathbf{E}, \mathbf{B})$] equals the response odd in $\mathbf{L}_0$ [i.e., $\underline{\sigma}(\mathcal{N}_0, \mathbf{L}_0, \mathbf{E}, \mathbf{B}) - \underline{\sigma}(\mathcal{N}_0, -\mathbf{L}_0, \mathbf{E}, \mathbf{B})$].

One can now expand $\underline{\sigma}(\mathcal{N}_0, \mathbf{L}_0, \mathbf{E}, \mathbf{B})$ with respect to the driving fields $\mathbf{E}$ and $\mathbf{B}$. To linear order and omitting $\mathcal{N}_0$ in the notation, we find that

$$\underline{\sigma}(\mathbf{L}_0, \mathbf{E}, \mathbf{B}) = \underline{\sigma}_0(\mathbf{L}_0) + \sum A_{\mathrm{e}k}(\mathbf{L}_0) E_k + \sum A_{\mathrm{m}k}(\mathbf{L}_0) B_k, \tag{32}$$

where the second-rank response tensors fulfill $A_{\mathrm{e}k}(\mathbf{L}_0) = -A_{\mathrm{e}k}(-\mathbf{L}_0)$ and $A_{\mathrm{m}k}(\mathbf{L}_0) = A_{\mathrm{m}k}(-\mathbf{L}_0)$. It follows that a response odd in $\mathbf{L}_0$ and linear in the driving field $F = (\mathbf{E}, \mathbf{B})$ can only arise from the electric field of the pump pulse. As $A_{\mathrm{e}k}(\mathbf{L}_0)$ is odd in $\mathbf{L}_0$, the linear electric response is zero for vanishing magnetic order ($\mathbf{L}_0 = 0$). This result is a direct consequence of the fact that inversion symmetry of Mn_2Au is solely broken by the antiferromagnetic order.

Point-group symmetries. We expand the tensors $A_{\mathrm{e}k}(\mathbf{L}_0)$ and $A_{\mathrm{m}k}(\mathbf{L}_0)$ in Eq. (32) up to second order in $\mathbf{L}_0$ to obtain

$$\sigma_{ij}^{(\mathrm{p})} = \sigma_{ij}^{0(\mathrm{p})} + \sum \delta_{ijk}^{(\mathrm{a})} B_k + \sum \beta_{ijkl}^{(\mathrm{p})} L_{0k} L_{0l} + \sum \chi_{ijkl}^{(\mathrm{a,s})} E_l L_{0k} + \sum \chi_{ijklm}^{(\mathrm{a})} B_l L_{0k} L_{0m}. \tag{33}$$

Here, the superscripts p, a and s indicate whether the given tensor is polar, axial and/or staggered. Eq. (33) implies that changes due to $\mathbf{E}$ are exclusively captured by the $\chi_{ijkl}^{(\mathrm{a,s})}$ term.

The 4/mmm point-symmetry group of the Mn_2Au crystal lattice imposes constraints on the form of the response tensors in Eq. (33).[25] Following Ref. [25], we choose a Cartesian frame with unit vectors $\mathbf{u}_1 \parallel [110]$, $\mathbf{u}_2 \parallel [\bar{1}10]$ and $\mathbf{u}_3 \parallel [001]$, where $\mathbf{u}_1$ and $\mathbf{u}_2$ coincide with the easy axes of the Néel vector $\boldsymbol{L}$. It is sufficient to consider the 3 generators $\bar{1}^{(\mathrm{s})}$, $2_{\mathbf{u}_2}^{(\mathrm{s})}$ and $4_{\mathbf{u}_3}$ of the 4/mmm group[26]: space inversion, 180° rotation about $\mathbf{u}_2$ and 90° rotation about $\mathbf{u}_3$. The presence of the superscript (s) indicates whether the operation is staggered or not. As our probe beam is normally incident on the Mn_2Au thin film, only the in-plane directions $\mathbf{u}_1$ and $\mathbf{u}_2$ matter, and the relevant conductivity-tensor elements are given by

$$\begin{pmatrix}\sigma_{11} & \sigma_{12}\\ \sigma_{21} & \sigma_{22}\end{pmatrix}(\mathbf{L}_0,\mathbf{E},\mathbf{B})$$
$$= \begin{pmatrix}\sigma_{11}^0 & 0\\ 0 & \sigma_{11}^0\end{pmatrix} + \begin{pmatrix}0 & \delta B_3\\ -\delta B_3 & 0\end{pmatrix} + \begin{pmatrix}\sum\beta_{1j}L_{0j}^2 & \beta_{66}L_{01}L_{02}\\ \beta_{66}L_{01}L_{02} & \beta_{12}L_{01}^2+\beta_{11}L_{02}^2+\beta_{13}L_{03}^2\end{pmatrix} + \begin{pmatrix}\chi_1E_1L_{02}+\chi_2E_2L_{01} & \chi_3E_1L_{01}+\chi_4E_2L_{02}+\chi_5E_3L_{03}\\ -\chi_4E_1L_{01}-\chi_3E_2L_{02}-\chi_5E_3L_{03} & -\chi_1E_2L_{01}-\chi_2E_1L_{02}\end{pmatrix}. \quad (34)$$

In the case that $\mathbf{E}$ and $\mathbf{B}$ are static, all coefficients can be further restricted by using Onsager relations. We do not make use of Onsager relations here because it is not straightforward to generalize them to time-varying (terahertz) fields. In Eq. (34), the first and third terms are symmetric under exchange of indices, the second term is antisymmetric, while the fourth term generally has mixed contributions. The last term of Eq. (33) does not show up as a fifth term in Eq. (34) because it contains components that include L_{03} and/or B_3, which vanish in our sample or experimental geometry (Fig. 1a).

Conductivity. From Eqs. (8) and (34), we can directly obtain $\sigma_{22}-\sigma_{11}$, σ_s and σ_a. By transformation into the laboratory frame and using $E_3=E_z=0$ and $B_3=B_z=0$, we arrive at

$$\begin{aligned}\sigma_{22}-\sigma_{11} &= aL_{0\parallel}^2\cos(2\varphi_{\mathbf{L}_0})+\eta L_{0\parallel}E(t)\sin(\varphi_\mathbf{E}-\varphi_\mathrm{s}+\varphi_{\mathbf{L}_0}),\\ \sigma_\mathrm{s} &= bL_{0\parallel}^2\sin(2\varphi_{\mathbf{L}_0})+\kappa L_{0\parallel}E(t)\cos(\varphi_\mathbf{E}-\varphi_\mathrm{s}+\varphi_{\mathbf{L}_0}),\\ \sigma_\mathrm{a} &= \mu L_{0\parallel}E(t)\cos(\varphi_\mathbf{E}-\varphi_\mathrm{s}-\varphi_{\mathbf{L}_0}).\end{aligned} \quad (35)$$

By definition, the coefficients $a=\beta_{12}-\beta_{11}$, $b=\beta_{66}/2$, $\eta=-(\chi_1+\chi_2)$, $\kappa=(\chi_3-\chi_4)/2$ and $\mu=-(\chi_3+\chi_4)/2$ are independent of $\mathbf{L}_0$, $\mathbf{E}$ and $\mathbf{B}$. In our experiment, $\varphi_{\mathbf{L}_0}-\varphi_\mathrm{s}$, is an integer multiple of 90° (Fig. 1a). In a single domain, we have $\mathbf{L}_0\parallel\mathbf{u}_1$ or $\mathbf{L}_0\parallel\mathbf{u}_2$, which would simplify Eq. (35) further. The symmetric off-diagonal σ_s contains a term bilinear in $\mathbf{E}$ and $\mathbf{L}_0$ and is, thus, consistent with the observed signals shown in Fig. 2.

Note that, in general, terms $\propto \eta,\kappa$ and μ capture pump-induced changes in both magnetic and non-magnetic observables. Therefore, one may write $\eta=\eta_\mathcal{S}+\eta_\mathcal{N}$ (and κ and μ analogous), where $\eta_\mathcal{S}$ and $\eta_\mathcal{N}$ is the contribution from pure spin and non-spin degrees of freedom, respectively.

Pump field: implicit treatment

Instead of accounting for the pump field explicitly, we are often rather interested in its effect on the spin degrees of freedom. Thus, the pump field $(\mathbf{E},\mathbf{B})$ is implicitly captured by the changes it imposes on the magnetic order and on the non-spin degrees of freedom. We, thus, choose $X=(\mathcal{N},\mathcal{S})$ with $\mathcal{S}=(\mathbf{M},\mathbf{L})$ [see Eq. (28)].

In general, the presence of the pump field lowers the sample symmetry and, in particular, breaks the inversion symmetry of $\mathcal{N}$. Therefore, we cannot apply the steps leading to Eq. (31) above. However, as we are only interested in effects linear in the pump field and, thus, $\Delta\mathcal{N}$ and $\Delta\mathcal{S}$, we can write the pump-induced change in the probe conductivity as $\Delta\underline{\sigma}=\Delta_\mathcal{N}\underline{\sigma}+\Delta_\mathcal{S}\underline{\sigma}$, where $\Delta_\mathcal{N}\underline{\sigma}=\underline{\sigma}(\mathcal{N},\mathcal{S}_0)-\underline{\sigma}_0$ and $\Delta_\mathcal{S}\underline{\sigma}=\underline{\sigma}(\mathcal{N}_0,\mathcal{S})-\underline{\sigma}_0$. As a consequence, we have

$$\underline{\sigma}(\mathcal{N},\mathbf{M},\mathbf{L})=\underline{\sigma}(\mathcal{N}_0,\mathbf{M},\mathbf{L})+\Delta_\mathcal{N}\underline{\sigma} \quad (36)$$

up to first order in the driving field.

Magnetic contribution. The term $\underline{\sigma}(\mathcal{N}_0,\mathbf{M},\mathbf{L})$ in Eq. (36) describes true spin dynamics because its time dependence arises solely from the spin degrees of freedom. As $\mathcal{N}_0$ has all symmetries of the equilibrium sample, we can derive constraining relationships for $\underline{\sigma}(\mathcal{N}_0,\mathbf{M},\mathbf{L})$ precisely as for the equilibrium case. Analogous to Eq. (31), space inversion leads to

$$\underline{\sigma}(\mathcal{N}_0,\mathbf{M},\mathbf{L})=\underline{\sigma}(\mathcal{N}_0,\mathbf{M},-\mathbf{L}). \quad (37)$$

To further determine the impact of magnetic order on the optical probe, we expand $\underline{\sigma}(\mathcal{N}_0,\mathbf{M},\mathbf{L})$ up to second order in $(\mathbf{M},\mathbf{L})$ and obtain

$$\sigma_{ij}(\mathcal{N}_0,\mathbf{M},\mathbf{L})=\sigma_{ij}^0(N_0,0,0)+\sum\alpha_{ijk}M_k+\sum\beta_{ijkm}L_kL_m+\sum\gamma_{ijkm}M_kM_m. \quad (38)$$

Due to Eq. (37), the linear term is independent of $\mathbf{L}$, and the quadratic term has no mixed $\mathbf{M}$-$\mathbf{L}$ contribution. The last term is omitted because it is small ($\mathbf{M}_0 = 0$). Finally, the Mn_2Au point group restricts the conductivity tensor in the film plane to

$$\begin{pmatrix} \sigma_{11} & \sigma_{12} \\ \sigma_{21} & \sigma_{22} \end{pmatrix} (N_0, \mathbf{M}, \mathbf{L}) = \begin{pmatrix} \sigma_{11}^0 & 0 \\ 0 & \sigma_{11}^0 \end{pmatrix} + \begin{pmatrix} 0 & \alpha M_3 \\ -\alpha M_3 & 0 \end{pmatrix} + \begin{pmatrix} \sum \beta_{1j} L_j^2 & \beta_{66} L_1 L_2 \\ \beta_{66} L_1 L_2 & \beta_{12} L_1^2 + \beta_{11} L_2^2 + \beta_{13} L_3^2 \end{pmatrix}. \quad (39)$$

The first and third terms in Eq. (39) are similar to the pump-independent contributions in Eq. (34), since β_{ijkm} is only restricted by crystallographic symmetry. The second term corresponds to a Faraday rotation due to an induced magnetization in the antiferromagnet. One can also see that the symmetric off-diagonal term $\sigma_\mathrm{s} = \beta_{66} L_1 L_2$ only depends on the Néel vector.

Non-magnetic contribution. With regard to $\Delta_\mathcal{N} \underline{\sigma}$, we note that we are not interested in further specification of the pump-induced changes $\Delta\mathcal{N}$ in the non-spin degrees of freedom. Therefore, we again express $\Delta_\mathcal{N} \underline{\sigma}$ by the driving pump field to linear order. The resulting conductivity matrix has the same form as the pump-field-dependent terms in Eq. (34), just with different coefficients, namely $\eta_\mathcal{N}$, $\kappa_\mathcal{N}$ and $\mu_\mathcal{N}$.

Conductivity. By combination of Eqs. (8) and (39) and $\Delta_\mathcal{N} \underline{\sigma}$ and by transformation into the lab system, we obtain

$$\begin{aligned} \sigma_{22} - \sigma_{11} &= a L_\parallel^2(t) \cos[2\varphi_\mathbf{L}(t)] + \eta_\mathcal{N} L_{0\parallel} E(t) \sin(\varphi_\mathbf{E} - \varphi_\mathrm{s} + \varphi_{\mathbf{L}_0}), \\ \sigma_\mathrm{s} &= b L_\parallel^2(t) \sin[2\varphi_\mathbf{L}(t)] + \kappa_\mathcal{N} L_{0\parallel} E(t) \cos(\varphi_\mathbf{E} - \varphi_\mathrm{s} + \varphi_{\mathbf{L}_0}), \\ \sigma_\mathrm{a} &= c M_z(t) + \mu_\mathcal{N} L_{0\parallel} E(t) \cos(\varphi_\mathbf{E} - \varphi_\mathrm{s} - \varphi_{\mathbf{L}_0}). \end{aligned} \quad (40)$$

The first term on the right-hand side of each Eq. (40) monitors true spin dynamics. It is analogous to the first term in each Eq. (35), where the time-dependent values $\mathbf{M}$ and $\mathbf{L}$ are just substituted by their time-independent counterparts $\mathbf{M}_0 = 0$ and $\mathbf{L}_0$. The second term in each Eq. (40) arises from pump-induced variations in the non-spin degrees of freedom. It has exactly the same structure as the second term in Eq. (35), just with the coefficients η, κ and μ substituted by $\eta_\mathcal{N}$, $\kappa_\mathcal{N}$ and $\mu_\mathcal{N}$

Comparison of both treatments

As Eq. (40) connects the measured signal to the spin dynamics in a more direct way than Eq. (35), we use it for the analysis of the observed signals in the main text. However, the 2 approaches complement each other in the discussion of symmetries, and it is useful compare them in more detail.

Magnetic dynamics. Comparison of Eqs. (40) and (35) allows us to connect the pump-induced changes of $(\mathbf{M}, \mathbf{L})$ to the driving terahertz pump field $(\mathbf{E}, \mathbf{B})$ by substituting $\mathbf{M} = \mathbf{M}_0 + \Delta\mathbf{M} = \Delta\mathbf{M}$ and $\mathbf{L} = \mathbf{L}_0 + \Delta\mathbf{L}$ into Eq. (40). We focus on the off-diagonal symmetric magnetic part of the conductivity and obtain

$$\sigma_\mathrm{s}(\mathcal{N}_0, \mathbf{M}, \mathbf{L}) = \beta_{66}(L_{01} L_{02} + L_{01}\Delta L_2 + L_{02}\Delta L_1) + \mathcal{O}(\Delta\mathbf{L}^2). \quad (41)$$

On the other hand, we can also separate Eq. (35) explicitly into magnetic and non-magnetic parts, obtaining in particular

$$\sigma_\mathrm{s}(\mathbf{L}_0, \mathbf{E}, \mathbf{B}) = \beta_{66} L_{01} L_{02} + (\kappa_\mathcal{S} + \kappa_\mathcal{N})(L_{01} E_1 - L_{02} E_2). \quad (42)$$

Consistence of Eqs. (41) and (42) is established if $\Delta L_2 = (\kappa_\mathcal{S}/\beta_{66}) E_1$ and $\Delta L_1 = -(\kappa_\mathcal{S}/\beta_{66}) E_2$. These relationships are compatible with the microscopic scenario detailed in Fig. 3a of the main text, given the Rashba-like symmetry of the staggered spin-orbit fields and the exchange torques[27]. An analogous treatment is possible for other contributions, which are, however, not relevant for the signals observed here.

Nonmagnetic dynamics. As the pump-dependent part in Eq. (35) generally has a spin and non-spin part by $\kappa = \kappa_\mathcal{S} + \kappa_\mathcal{N}$, the signal due to the pump-induced changes in the non-spin degrees of freedom $\propto \kappa_\mathcal{N}$ has the same dependence on $\varphi_\mathbf{E}$, $\varphi_{\mathbf{L}_0}$ and φ_s as the signal $\propto \kappa_\mathcal{S}$ due to the true spin dynamics. Consequently, these signals cannot be separated based on variation of $\varphi_\mathbf{E}$, $\varphi_{\mathbf{L}_0}$ and φ_s.

Supplementary references

1. Sajadi M., Wolf M. & Kampfrath T. Terahertz-field-induced optical birefringence in common window and substrate materials. *Opt. Express* **23**, 28985-28992 (2015).
2. Balos V., Wolf M., Kovalev S. & Sajadi M. Optical rectification and electro-optic sampling in quartz. *Opt. Express* **31**, 13317 (2023).
3. Leitenstorfer A., Hunsche S., Shah J., Nuss M. C. & Knox W. H. Detectors and sources for ultrabroadband electro-optic sampling: Experiment and theory. *Appl. Phys. Lett.* **74**, 1516-1518 (1999).
4. Kampfrath T., Nötzold J. & Wolf M. Sampling of broadband terahertz pulses with thick electro-optic crystals. *Appl. Phys. Lett.* **90**, (2007).
5. Gomonay O. & Bossini D. Linear and nonlinear spin dynamics in multi-domain magnetoelastic antiferromagnets. *J. Phys. D: Appl. Phys.* **54**, 374004 (2021).
6. Bodnar S. Y. et al. Magnetoresistance Effects in the Metallic Antiferromagnet Mn2Au. *Phys. Rev. Appl.* **14**, (2020).
7. Bommanaboyena S. P. et al. Readout of an antiferromagnetic spintronics system by strong exchange coupling of Mn2Au and Permalloy. *Nat. Commun.* **12**, 6539 (2021).
8. Jin Z. et al. Ultrafast electron transport in metallic antiferromagnetic Mn_2Au thin films probed by terahertz spectroscopy. *Phys. Rev. B* **102**, 014438 (2020).
9. Wang J. et al. The structural, elastic, phonon, thermal and electronic properties of MnX (X=Ni, Pd and Pt) alloys: First-principles calculations. *J. Magn. Magn. Mater.* **333**, 93-99 (2013).
10. Kang K., Cahill D. G. & Schleife A. Phonon, electron, and magnon excitations in antiferromagnetic L10-type MnPt. *Phys. Rev. B* **107**, (2023).
11. Zhao C., Kang K., Neuefeind J. C., Schleife A. & Shoemaker D. P. In-plane magnetic structure and exchange interactions in the high-temperature antiferromagnet Cr2Al. *Phys. Rev. Mater.* **5**, 084411 (2021).
12. Gomonay O., Jungwirth T. & Sinova J. Narrow-band tunable terahertz detector in antiferromagnets via staggered-field and antidamping torques. *Phys. Rev. B* **98**, 104430 (2018).
13. Kampfrath T. et al. Coherent terahertz control of antiferromagnetic spin waves. *Nat. Photon.* **5**, 31-34 (2011).
14. Mashkovich E. A. et al. Terahertz Optomagnetism: Nonlinear THz Excitation of GHz Spin Waves in Antiferromagnetic $FeBO_3$. *Phys. Rev. Lett.* **123**, 157202 (2019).
15. Mondal R., Donges A., Ritzmann U., Oppeneer P. M. & Nowak U. Terahertz spin dynamics driven by a field-derivative torque. *Phys. Rev. B* **100**, 060409 (2019).
16. Barthem V. M. T. S., Colin C. V., Mayaffre H., Julien M. H. & Givord D. Revealing the properties of Mn2Au for antiferromagnetic spintronics. *Nat. Commun.* **4**, 2892 (2013).
17. Arana M. et al. Observation of magnons in Mn2Au films by inelastic Brillouin and Raman light scattering. *Appl. Phys. Lett.* **111**, 192409 (2017).
18. Chekhov A. L. et al. Ultrafast Demagnetization of Iron Induced by Optical versus Terahertz Pulses. *Phys. Rev. X* **11**, 041055 (2021).
19. Nádvorník L. et al. Broadband Terahertz Probes of Anisotropic Magnetoresistance Disentangle Extrinsic and Intrinsic Contributions. *Phys. Rev. X* **11**, (2021).
20. Kim Y., Yi M., Kim B. G. & Ahn J. Investigation of THz birefringence measurement and calculation in Al2O3 and LiNbO3. *Appl. Opt.* **50**, 2906-2910 (2011).
21. Grischkowsky D., Keiding S., Van Exter M. & Fattinger C. Far-infrared time-domain spectroscopy with terahertz beams of dielectrics and semiconductors. *J. Opt. Soc. Am. B* **7**, 2006 (1990).
22. Passler N. C. & Paarmann A. Generalized 4 × 4 matrix formalism for light propagation in anisotropic stratified media: study of surface phonon polaritons in polar dielectric heterostructures. *J. Opt. Soc. Am. B* **34**, 2128 (2017).
23. Touloukian Y. S. & Buyco E. H. Thermophysical properties of matter-the TPRC data series. Volume 4. Specific heat - metallic elements and alloys.). Purdue Univ., Lafayette, IN (United States). Thermophysical and Electronic Properties Information Center (1971).
24. Reimers S. et al. Current-driven writing process in antiferromagnetic Mn2Au for memory applications. *Nat. Commun.* **14**, (2023).
25. Freimuth F., Blügel S. & Mokrousov Y. Laser-induced torques in metallic antiferromagnets. *Phys. Rev. B* **103**, 174429 (2021).
26. Birss R. R. *Symmetry and magnetism* (North-Holland Pub. Co., Amsterdam, 1966).
27. Železný J. et al. Spin-orbit torques in locally and globally noncentrosymmetric crystals: Antiferromagnets and ferromagnets. *Phys. Rev. B* **95**, 014403 (2017).
